# Over-the-scope clip and self-expandable metal stent: a comprehensive treatment for failed peroral endoscopic myotomy and fibrosis complications in idiopathic achalasia

**DOI:** 10.1055/a-1953-7164

**Published:** 2022-10-21

**Authors:** Valeria D’Ovidio, Alessandra Cesarini, Daniele Lisi, Danilo Badiali, Marco E. Bazuro

**Affiliations:** 1 Gastrointestinal Endoscopy Unit, S. Eugenio Hospital, Rome, Italy; 2 Gastroenterological Unit, Umberto I Hospital, University La Sapienza, Rome, Italy

Achalasia is a rare esophageal motility disorder that affects both sexes and all ages. Peroral endoscopic myotomy (POEM) has been reported as an optional treatment since 2010 1 .

Frequently associated adverse events include pneumoperitoneum, pneumomediastinum, and pneumothorax, which are usually asymptomatic and managed conservatively 2 3 . Perforation, bleeding, mediastinitis, and peritonitis rarely occur and are often symptomatic. Mucosal injuries (including dehiscence, ulcer, and ischemia) do not alter the post-procedural course 4 .

A 31-year-old man, affected by idiopathic achalasia, was admitted as an outpatient to our Gastroenterological Unit owing to symptom recurrence and weight loss (Eckardt score 8). Two years before, he had undergone POEM, which was complicated by pneumomediastinum and ischemic damage of the distal esophagus with residual fibrosis. An esophagogram revealed a dilated esophagus (maximum diameter 60 mm) and supracardial stricture extending 15 mm above ( Video 1 ). His dysphagia was most likely worsened by post-POEM complications.

We planned to place a fully covered removable metal stent (SEMS; 60 × 27 mm) to achieve progressive, effective dilation of the distal esophagus ( Video 1 ). The SEMS would be firmly fixed by means of an over-the-scope (OTS) clip. This novel OTS clip device has demonstrated success in reducing SEMS migration even in benign diseases 5 .

**Video 1** Placement of a self-expandable metal stent secured by an over-the-scope clip for comprehensive treatment of achalasia after failed peroral endoscopic myotomy with fibrosis complications.


The SEMS was released 10 mm above the post-POEM stricture, 25 mm above the cardia, and fixed by means of the OTS clip. The patient was discharged uneventfully on the same day. An alternative approach (Heller myotomy) was ready to be employed in case of failure.

At the 4-week follow-up, dysphagia had improved and the patient had gained weight. Both the OTS clip and the SEMS were removed using the remOVE device (Ovesco Endoscopy AG, Tübingen, Germany) ( Video 1 ). The patient was discharged uneventfully 12 hours later.

After a further 4 weeks, an esophagogram revealed a significant reduction in the esophagus dilation and the patient’s clinical condition had significantly improved (Eckardt score 0) ( Video 1 ).

Endoscopy_UCTN_Code_CPL_1AH_2AD

## Notes

Endoscopy E-Videos

https://eref.thieme.de/e-videos
*Endoscopy E-Videos* is an open access online section, reporting on interesting cases and new techniques in gastroenterological endoscopy. All papers include a high quality video and all contributions are freely accessible online. Processing charges apply (currently EUR 375), discounts and wavers acc. to HINARI are available. This section has its own submission website at https://mc.manuscriptcentral.com/e-videos
